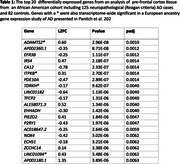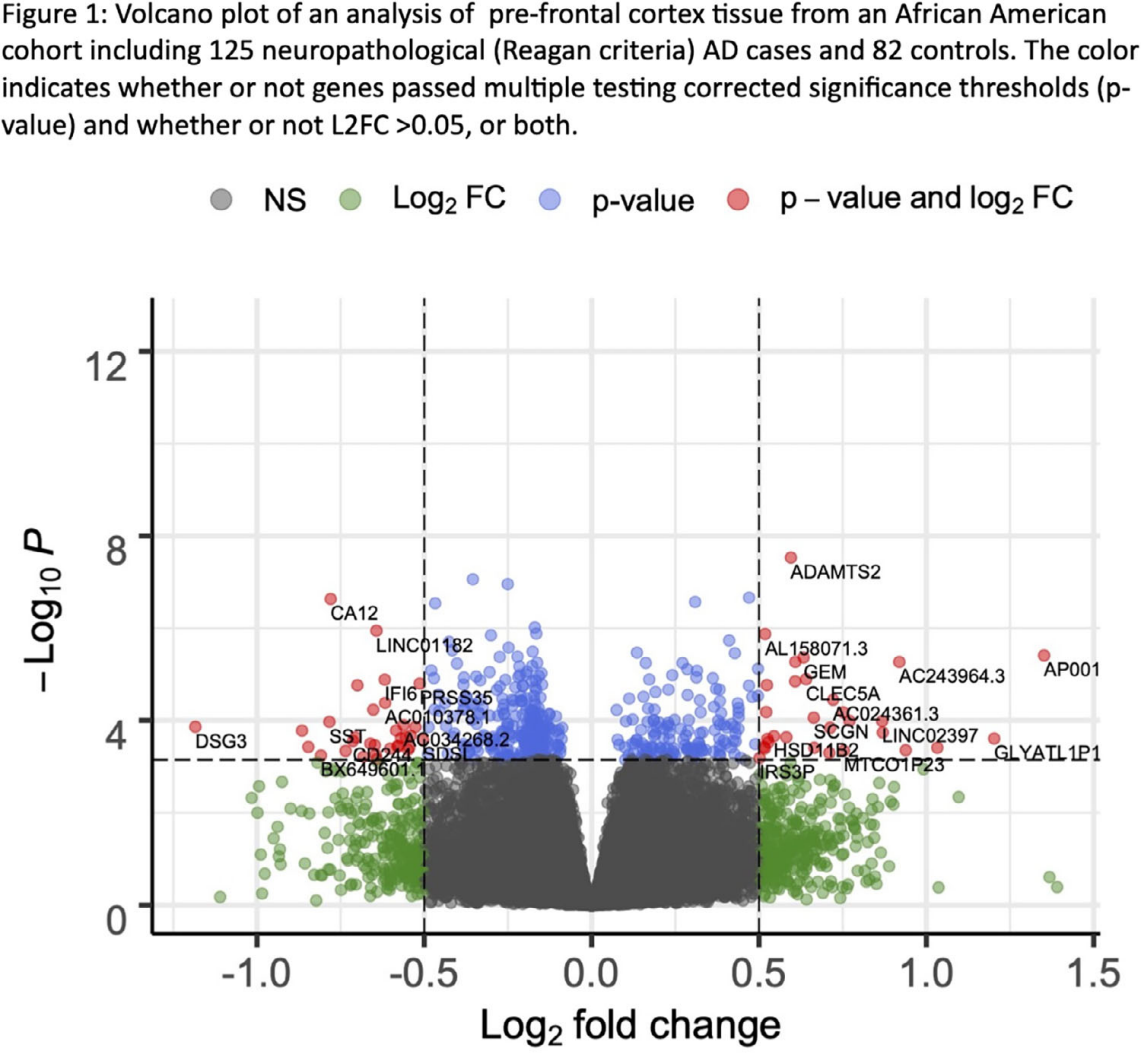# Identification of differentially expressed genes in post‐mortem tissue from African American Alzheimer disease cases and controls

**DOI:** 10.1002/alz.091575

**Published:** 2025-01-03

**Authors:** Mark W. Logue, Adam Thomas Labadorf, Nicholas K O'Neill, Dennis W. Dickson, Margaret E Flanagan, Erin E. Franklin, Matt P. Frosch, Marla Gearing, Lee‐Way Jin, Julia Kofler, Eddie B Lee, Richard Mayeux, Ann C. McKee, Carol A Miller, Melissa E. Murray, Peter T Nelson, Richard J. Perrin, Julie A. Schneider, Thor D. Stein, Andrew F Teich, Juan C Troncoso, Shih‐Hsiu Jerry Wang, Jesse Mez, Lindsay A. Farrer

**Affiliations:** ^1^ Boston University Chobanian & Avedisian School of Medicine, Boston, MA USA; ^2^ National Center for PTSD, VA Boston Healthcare System, Boston, MA USA; ^3^ Boston University, Boston, MA USA; ^4^ Department of Neuroscience, Mayo Clinic, Jacksonville, FL USA; ^5^ Northwestern University Feinberg School of Medicine, Chicago, IL USA; ^6^ Washington University in St. Louis School of Medicine, St. Louis, MO USA; ^7^ Knight Alzheimer Disease Research Center, Saint Louis, MO USA; ^8^ Neuropathology Service, C.S. Kubik Laboratory for Neuropathology, Massachusetts General Hospital, Harvard Medical School, Boston, MA USA; ^9^ Emory University, Atlanta, GA USA; ^10^ Emory University School of Medicine, Atlanta, GA USA; ^11^ University of California Davis Medical Center, Davis, CA USA; ^12^ University of Pittsburgh, Pittsburgh, PA USA; ^13^ University of Pennsylvania, Philadelphia, PA USA; ^14^ Columbia University Medical Center, New York, NY USA; ^15^ VA Bedford Healthcare System, Bedford, MA USA; ^16^ Alzheimer’s Disease Research Center, Keck School of Medicine, University of Southern California, Los Angeles, CA USA; ^17^ University of Southern California, Los Angeles, CA USA; ^18^ Mayo Clinic, Jacksonville, FL USA; ^19^ University of Kentucky, Lexington, KY USA; ^20^ The Charles F. and Joanne Knight Alzheimer Disease Research Center, Washington University, St. Louis, MO USA; ^21^ Washington University in St. Louis, St. Louis, MO USA; ^22^ Rush University Medical Center, Chicago, IL USA; ^23^ Department of Pathology and Laboratory Medicine, Boston University Chobanian & Avedisian School of Medicine, Boston, MA USA; ^24^ VA Boston Healthcare System, Boston, MA USA; ^25^ Taub Institute for Research on Alzheimer’s Disease and the Aging Brain, New York, NY USA; ^26^ Department of Neurology, New York, NY USA; ^27^ Columbia University Irving Medical Center, New York, NY USA; ^28^ Department of Pathology and Cell Biology, New York, NY USA; ^29^ Johns Hopkins University School of Medicine, Baltimore, MD USA; ^30^ Duke University Medical Center, Durham, NC USA; ^31^ Boston University Alzheimer’s Disease Research Center, Boston University Chobanian & Avedisian School of Medicine, Boston, MA USA; ^32^ Department of Medicine (Biomedical Genetics), Boston University Chobanian & Avedisian School of Medicine, Boston, MA USA; ^33^ Alzheimer’s Disease Research Center, Boston University Chobanian & Avedisian School of Medicine, Boston, MA USA; ^34^ Department of Ophthalmology, Boston University Chobanian & Avedisian School of Medicine, Boston, MA USA; ^35^ Department of Neurology, Boston University Chobanian & Avedisian School of Medicine, Boston, MA USA; ^36^ Department of Epidemiology, Boston University School of Public Health, Boston, MA USA; ^37^ Department of Biostatistics, Boston University School of Public Health, Boston, MA USA

## Abstract

**Background:**

Although the rate of Alzheimer’s disease (AD) in African‐ancestry (AA) Americans is higher than that of persons from European‐ancestry (EA) populations, AA participants have been underrepresented in AD neuropathological studies.

**Method:**

Utilizing the AD Research Centers (ADRC) infrastructure, we obtained AA donor pre‐frontal cortex (PFC) tissue from brain repositories of 12 ADRC and generated bulk RNA sequencing (RNA‐seq) data for 179 samples that met QC and inclusion criteria. Previously generated PFC RNAseq data were obtained for 28 additional AA donors from the Columbia University ADRC. Differential gene expression was evaluated among 125 donors with a neuropathological diagnosis of AD (NIA‐Reagan intermediate or high likelihood) and 82 neuropathologically confirmed controls using regression models including covariates for age at death, sex, cell‐type frequencies, and RNA integrity number (RIN) calculated with Limma. FDR‐corrected p‐values (p_adj_) were calculated to control for the 33,611 genes examined.

**Result:**

A total of 482 genes surpassed the multiple‐testing threshold. The most significant, *ADAMTS2* (p = 2.96 × 10^‐8^, p_adj_ = 0.001), showed increased expression in AD cases (see Table/Figure). We note that *ADAMTS2* was differentially expressed in a prior EA study of neuropathologically confirmed AD cases and controls (Panitch et al. *Molecular Psychiatry* 2021). Additionally, a recent analysis of cognitive resilience in EA neuropathological AD cases identified a strong association with *ADAMTS2* (See Li et al. AAIC2024 abstract). Of the differentially expressed genes observed in the Panitch et al. EA study, 385 (35%) were nominally significant,65 (5.8%) were corrected significant, and most (89%) of these genes had the same effect direction in the AA cohort. Some of the observed associations appear to be AA specific (e.g., *EFR3B*, *IRS4*, and *CA12*; see Table). Additionally, we found nominally significant (p<0.05) associations with expression of *APOE* (Log2 fold change [L2FC = ‐0.20, p = 0.012) and several other established AD genes including *SORL1* (L2FC = ‐0.10, p = 0.014) and *IGF1R*(L2FC = 0.11, p = 0.0013).

**Conclusion:**

This largest‐ever (to our knowledge) gene expression study of AD in postmortem brain tissue from AA donors implicates many more genes as having a role in AD in this population than previously identified in genome‐wide association studies and provides insight into trans‐ancestry differences risk for AD.